# Impact of Cross-Validation on Machine Learning Models for Early Detection of Intrauterine Fetal Demise

**DOI:** 10.3390/diagnostics13101692

**Published:** 2023-05-10

**Authors:** Jayakumar Kaliappan, Apoorva Reddy Bagepalli, Shubh Almal, Rishabh Mishra, Yuh-Chung Hu, Kathiravan Srinivasan

**Affiliations:** 1 School of Computer Science and Engineering, Vellore Institute of Technology, Vellore 632014, India; jayakumar.k@vit.ac.in (J.K.); apoorva.bagepalli2019@vitstudent.ac.in (A.R.B.); shubh.almal2019@vitstudent.ac.in (S.A.); rishabh.mishra2019@vitstudent.ac.in (R.M.); kathiravan.srinivasan@vit.ac.in (K.S.); 2 Department of Mechanical and Electromechanical Engineering, National ILan University, Yilan 26047, Taiwan

**Keywords:** CTG (Cardiotocography) data, fetal health classification, machine learning, cross fold validation, explainable AI

## Abstract

Intrauterine fetal demise in women during pregnancy is a major contributing factor in prenatal mortality and is a major global issue in developing and underdeveloped countries. When an unborn fetus passes away in the womb during the 20th week of pregnancy or later, early detection of the fetus can help reduce the chances of intrauterine fetal demise. Machine learning models such as Decision Trees, Random Forest, SVM Classifier, KNN, Gaussian Naïve Bayes, Adaboost, Gradient Boosting, Voting Classifier, and Neural Networks are trained to determine whether the fetal health is Normal, Suspect, or Pathological. This work uses 22 features related to fetal heart rate obtained from the Cardiotocogram (CTG) clinical procedure for 2126 patients. Our paper focuses on applying various cross-validation techniques, namely, K-Fold, Hold-Out, Leave-One-Out, Leave-P-Out, Monte Carlo, Stratified K-fold, and Repeated K-fold, on the above ML algorithms to enhance them and determine the best performing algorithm. We conducted exploratory data analysis to obtain detailed inferences on the features. Gradient Boosting and Voting Classifier achieved 99% accuracy after applying cross-validation techniques. The dataset used has the dimension of 2126 × 22, and the label is multiclass classified as Normal, Suspect, and Pathological condition. Apart from incorporating cross-validation strategies on several machine learning algorithms, the research paper focuses on Blackbox evaluation, which is an Interpretable Machine Learning Technique used to understand the underlying working mechanism of each model and the means by which it picks features to train and predict values.

## 1. Introduction

Women go through a lot during their pregnancy. It is a very vital stage in a woman’s life, and the happiness brought by a newborn child is immeasurable at times. However, there are instances where a fetal demise occurs in the womb. Numerous causes, including intrapartum difficulties, hypertension, diabetes, infection, congenital and genetic abnormalities, and placental malfunction, contribute to such events [1,2]. According to a study conducted on 6942 deliveries, 250 intrauterine fetal deaths were reported. Anaemia, pregnancy-induced hypertension, illiteracy, and low socioeconomic position were the leading causes of these fetal fatalities. As per WHO statistics, globally, 2 million stillbirths occur every year, which is one death every 6 s. Among medical problems, the study discovered that hypertension and anemia were related to a greater likelihood of stillbirth. Pregnancy-induced hypertension was responsible for 19.6% of cases, antepartum hemorrhage was responsible for 12% of cases, labour trauma and stress were responsible for 34% of cases, maternal medical conditions were responsible for 12.8% of cases, fetal growth retardation was responsible for 5.2% of cases, congenital malformation was responsible for 8% of cases, prematurity was responsible for 2.8% of cases, and unknown etiology was responsible for 5.6% of cases [3]. Maintaining the proper health of the fetus plays an important part in the chance of survivability of the fetus. Cardiotocographic (CTG) data is utilized by medical professionals in order to predict the health of the fetus; however, due to the increasing frequency of examinations and the shortage of medical professionals, it becomes challenging to decide which patient should be granted priority or which patient needs proper intensive care. The purpose of this study is to iterate over different Machine Learning (ML) algorithms to find the optimal ML algorithm among Adaboost, Decision Tree, Gaussian Naïve Bayes, Gradient Boosting, K-Nearest Neighbor algorithm (KNN), Random Forest, Support Vector Machine (SVM) Classifier, Voting Classifier, and Feed Forward Network by applying different Cross-Validation techniques in order to provide an accurate prediction on the respective patients so that the medical professionals can have a chance to obtain early diagnostics results that could help save the patient’s unborn child, as well as save resources that would have otherwise been required while diagnosing all of their patients.

## 2. Related Works

Dilip Kumar Sharma [3] worked on Support Vector Machine, Random Forest, Multilayer Perceptron, and K-Nearest Neighbors to predict fetal health using data from CTG [1]. They discovered that fetal heart rate deceleration is an important marker in determining health status through Correlation Analysis and Regression Analysis. Nabillah Rahmayanti et al. performed a study that employed deep learning techniques to extract high-level features from a dataset in order to categorize fetal health using CTG data. Fetal health was categorized using a number of different machine-learning algorithms, such as Artificial Neural Networks (ANN), K-Nearest Neighbor algorithm (KNN), Light Gradient Boosting Machine (LGBM), Long Short-Term Memory (LSTM), Random Forest (RF), Support Vector Machine (SVM), and Extreme Gradient Boosting (XGB) [2]. Jiaming L Xiaoxiang Liu deployed twelve distinct machine learning models that were trained on the CTG dataset. The Blender model was constructed using the soft voting integration approach from the top four models, and it was contrasted with the stacking model. It fared quite well in tests of other classification models [4]. Ilias Tougui et al. investigated two cross-validation methodologies, subject-wise and record-wise techniques, to demonstrate the influence of machine learning algorithms such as Support Vector Machine and Random Forest trained to detect Parkinson’s disease [5]. To analyze and classify prenatal health problems, Jayashree Piri et al. suggested an association-based classification model. The author used several association rules to improve the classifier’s accuracy, obtaining an accuracy around 83–84% [6]. D. Tran, S. Cooke, P.J. Illingworth, and D.K. Gardner showed that deep learning has the same potential to improve clinical IVF by using the time-lapse video to predict fetal heart pregnancy. This study’s retrospective analysis has shown that IVY is a useful tool for predicting embryo implantation rate [7].

Anand Sontakke et al. classified cardiotocography signals using machine learning, performing 10-fold cross-validation and spot-checking on the dataset and analyzing the results [8]. The classification of the fetus state was carried out by Andrew Maranho et al. using the data from cardiotocography and machine learning algorithms. A lightgbm model that had been post-processed using cross-validation ensembling and adjusted with Gaussian process regression was used after a baseline random forest model [9]. Machine learning methods were used in the research of Md. Tamjid Rayhan, et al. on the automatic diagnosis of fetal health status using cardiotocography data [10]. In their study of five different machine learning algorithms, Eva Malacova and Sawitchaya Tippaya classified binary data as childbirth vs. live delivery. The classifiers included multilayer perceptron (MLP) neural networks, random forest, classification and regression trees (CART), and extreme gradient boosting (XGBoost) [11]. Naveen Reddy Navuluri researched fetal health prediction using classification techniques. In this research, four machine learning models were presented. The SVM model provided the best outcome with the highest accuracy among the four machine learning models [12]. Mario W.L Morerira et al. performed hypertensive disorder prediction in high-risk pregnancy groups using tree-based techniques ID3 and NBTree. Fmeasure, kappa static and ROC were used to assess its performance [13].

Efficient fetal acidosis detection using the relevant subset of features with sparse support vector machine classification was performed by Jiri Spilka et al. and it achieved better classification results [14]. The feature selection was carried out by Ragunath Dey et al. utilising a crowding distance-based multi-objective genetic algorithm (MOGA-CD). Using chi-square and ANOVA, the most important factors that determine the fetus’s health are assessed. The correlation matrix provides the connection strength between the characteristics and the target attribute [15]. Prakriti Dwivedi et al. classified the primary factors influencing fetal health status using cardiotocography measurements [16]. The fetal heart rate and uterine contractions are obtained respectively during cardiotocography, and the dataset was accessible at UCI. Three classification algorithms—Decision Tree (DT), Support Vector Machine (SVM), and Naïve Bayes—were applied to this dataset by Kanika Agrawal (NB) [17]. Using a convolutional neural network, Jianqiang Li et al. conducted research on the automatic classification of fetal heart rate. To categorize the fetal heart rate recordings and obtain the requisite accuracy, this study employed its own model as well as statistical techniques such as SVM and MLP [18]. Adem Kuzu and Yunus Santur conducted fetal health pattern classification using ensemble learning. Obstetricians can utilize CTG data to determine whether a fetus is healthy and when medical intervention is required. The goal of their study was to eliminate discrepancies by evaluating CTG data with neural networks [19].

### Motivation and Contribution

Various papers are associated with fetal health classification, but there has been no determination of the optimum model or optimum application of cross-validation to enhance the mechanism of any model. Apart from applying machine learning algorithms, there has been little or no work performed on the Blackbox evaluation of these algorithms. Thus, we were motivated to determine the optimum model and enhance the model’s outcome with various cross-validation techniques, as well as to evaluate the working model with Blackbox Evaluation.

This article focuses on:

- Investigating different cross-validation techniques applied.
- Performing Exploratory Data Analysis for inferences of data.
- Selecting the ideal model and using Blackbox evaluation to assess that model’s performance.

The literature survey is explained in Section 2, and in Section 3, the Methodology is described. The Exploratory Data Analysis and Blackbox evaluation are explained and illustrated in Section 4. Section 5 addresses the experimental result, and the conclusion is stated in Section 6.

## 3. Materials and Methods

### 3.1. Data Description 

The Cardiotocography Data Set was used [20]. This dataset has 2126 rows, 22 columns, and 3 class labels. The 3 classes of labels are Normal, Suspect, and Pathological condition. The list of the features and their descriptions are provided below. Fetal heartbeat and the mother’s uterine contraction are highly correlated with fetal health condition, and light deceleration is caused by uterine contractions compressing the fetal head. Uteroplacental deficit causes prolonged decelerations, which lead to a reduction in blood flow to the placenta that lowers the quantity of oxygen and nutrients provided to the fetus. As the dataset provided is imbalanced, we applied Random over Sampler to balance the dataset.

Feature Description:

1. Baseline Value—This feature describes the baseline fetal heart rate.
2. Acceleration—This feature indicates the number of accelerations per second.
3. Fetal Movement—This feature indicates the number of fetal movements per second.
4. Uterine Contraction—This feature indicates the number of uterine contractions per second.
5. Abnormal short-term variability—This feature provides the percentage of time with abnormal short-term variability.
6. Severe Deceleration—This feature presents the number of SDs per second.
7. Light Deceleration—This feature presents the number of LDs per second.
8. Prolonged Deceleration—This feature presents the number of PDs per second.

### 3.2. Preprocessing

The fetal dataset is made up of imbalance classes that were balanced with one of the sampling methods, such as over-sampling, to add minority cases to the dataset in order to establish balance, in which, existing minority examples are repeated or artificial minority examples are manufactured. It replicates comparable values by randomly picking samples with replacements for classes with a minority number until all classes are evenly balanced, improving the classification accuracy of every machine learning model.

### 3.3. Stages of Work Performed

Three stages of work were performed. They are:

1. Exploratory Data Analysis

**Figure 1:**
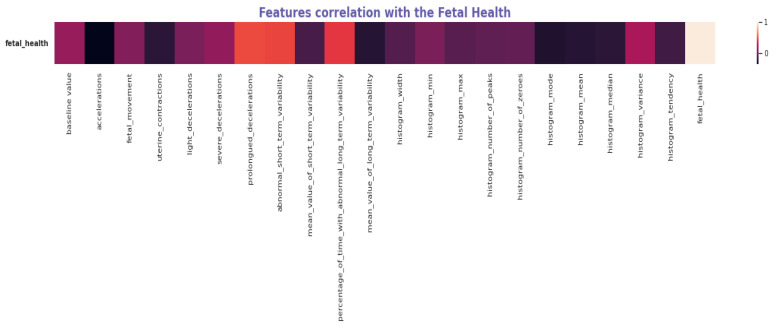
Feature Correlation with the fetal health label.

Exploratory Data Analysis (EDA) was performed to highlight the quality of the features that assist in defining fetal health conditions. Figure 1 shows the correlation of features with fetal health. as well as a varying range of values in features, helping to determine which are major deciding factors in determining the condition. High acceleration and lesser fetal movement have shown positive results in fetal health.

2. Classification

To determine the optimum classification algorithm among the applied algorithms, we classify the dataset with each algorithm and enhance the performance by tuning their hyper parameters, introducing different cross-validation techniques to the algorithms to generalize and avoid overfitting and underfitting issues during the training phase. Gradient Boosting and voting classifiers perform tremendously well after applying Monte Carlo Cross-validation Technique.

3. Blackbox Evaluation

Understanding the working mechanism of a model and interpreting it with a layman’s approach, Blackbox Evaluation such as SHAP and LIME was applied to understand the working of the models and the means by which the model decides which features determine the health condition of the fetus.

#### 3.3.1. Exploratory Data Analysis

**Figure 2:**
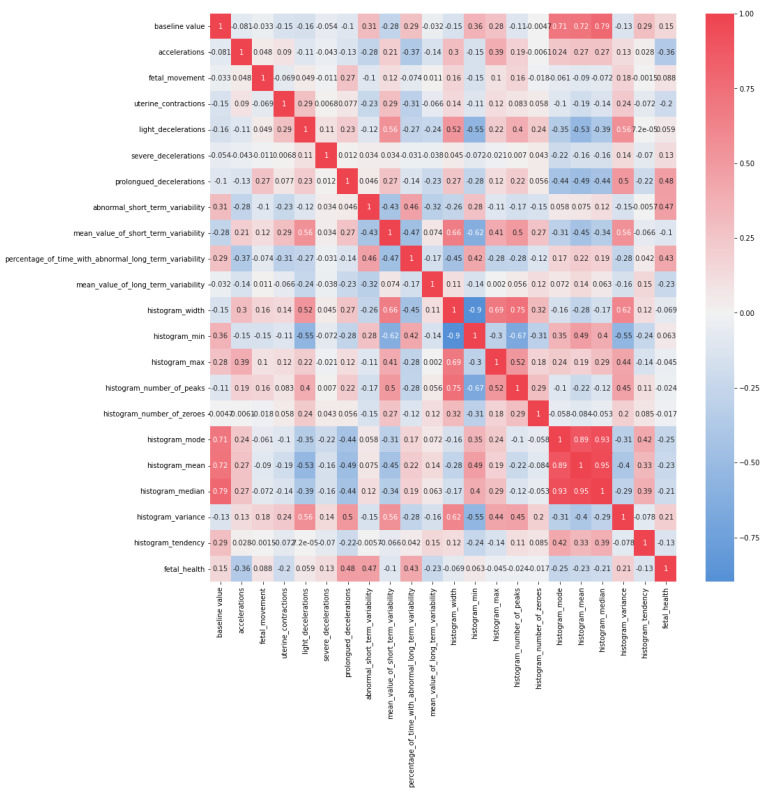
Correlation matrix on given fetal attributes.

Figure 2 depicts the association of several indicators and their importance in defining the state of fetal health. As shown in Figure 2 and Table 1, Acceleration, abnormal short-term variability, abnormal long-term variability percentage, long-term variability mean value, and prolonged decelerations have a high correlation with fetal health.

**Figure 3:**
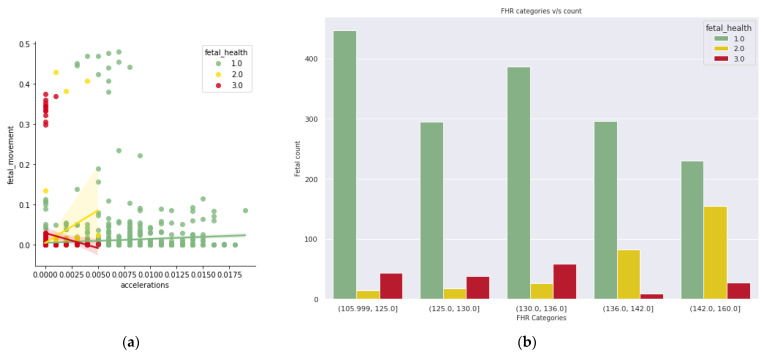
(**a**) Acceleration vs. Fetal Movement; (**b**) Count of Fetus with a range of Fetal Heart Range.

**Figure 4:**
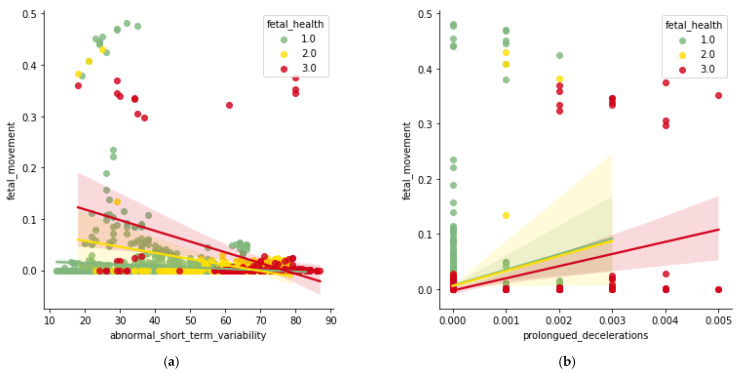
(**a**) Abnormal Short-term Variability vs. Fetal movement; (**b**) Prolonged Deceleration vs. Fetal movement.

In Figure 3a, with the higher range of acceleration and lesser number of fetal movements per second, the fetal health condition is detected as normal, whereas in the suspect and pathological condition, there is lesser acceleration and the fetal movement is arbitrary. In Figure 3b, in normal fetal condition, there is an arbitrary range, an incremental suspect condition with an increase in baseline rate, there is no trend identified with an increase in baseline rate, and there is no trend identified with the pathological condition and baseline rate. In Figure 4a, lesser fetal movement and arbitrary range in abnormality of short-term variability show the signs of normal fetal health condition, and lesser fetal movement and high-value range in abnormality of short-term variability shows that there is pathological as well as suspect condition. In Figure 4b, with less or no prolonged deceleration and arbitrary fetal movement, the fetal health is normal; however, increasing prolonged deceleration and arbitrary fetal movement show signs of a suspect and pathological condition of fetal health.

**Figure 5:**
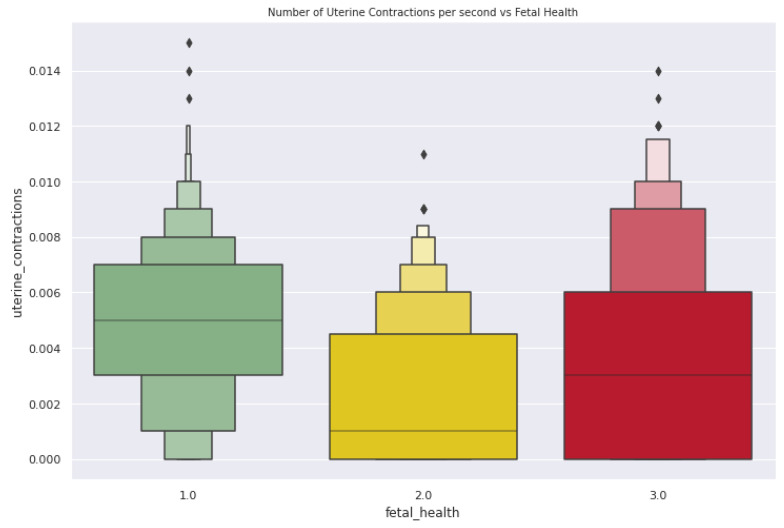
Number of uterine contractions per second vs. fetal health.

**Figure 6:**
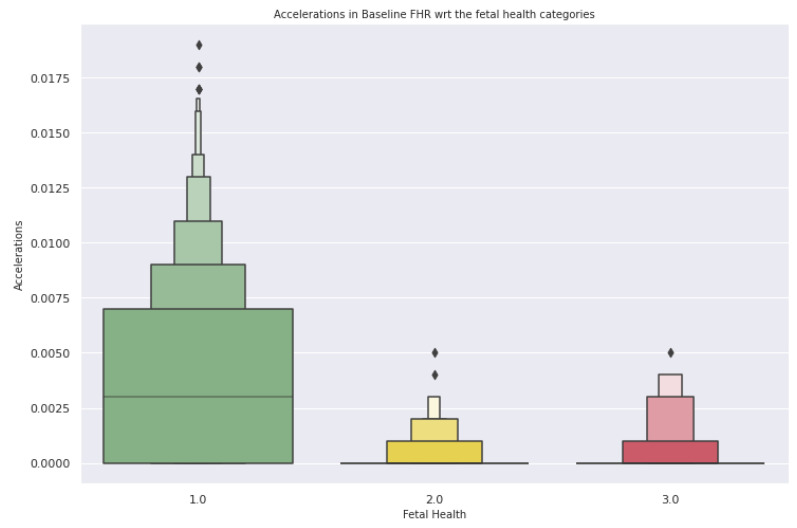
Acceleration of fetal heart rate vs. fetal health.

In Figure 5, the average uterine contractions are 0.005 contractions per second for a normal fetal condition, and about 0.002 contractions per second are identified in a suspect condition. In identifying a pathological condition, the average uterine contractions are 0.004 contractions per second. As shown in Figure 6, the plot determines how the acceleration of the fetal heart rate has a positive effect on fetal health.

From all the above figures, Acceleration in fetal heart rate showcases a healthy impact on the fetus, while the evidence of fewer fetal movements per second showcases the normal condition of fetal health. A gradual increase in prolonged deceleration of fetal heart rate has shown to be a cause of pathological conditions for the fetus.

#### 3.3.2. Classification

Identifying fetal abnormalities is a challenging task in the early stages, but it is now gradually becoming enhanced by various machine learning algorithms. There are several machine learning methods available for categorizing fetal health conditions [4]. To improve the performance of some classifiers, hyper parameter tuning was used. Hence, the evaluation of different machine learning algorithms along with various cross-validation techniques is applied, revealing the most effective algorithm in this paper.

**Figure 7:**
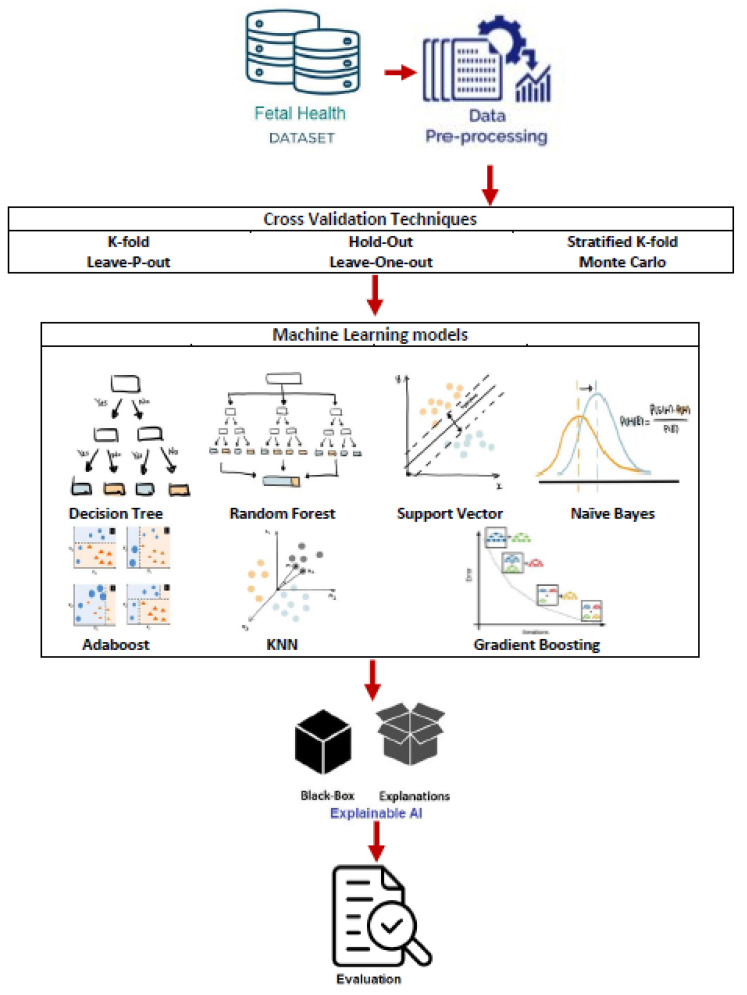
Proposed system architecture.

Figure 7 describes the architecture followed to develop the complete system. Extract and import the dataset from the source, pre-process the given data, i.e., applying an Oversampling approach for the imbalanced data set, standardize the features’ values, split the dataset randomly, train the data with different ML models and note the results for comparison, apply different Cross-validation techniques to each ML model, conduct an evaluation using the metrics, and determine the optimal ML model.

1. Decision Tree Classifier

Information is presented to decision trees in the form of trees for convenience. This could also be thought of as a collection of different rules. The main benefit of decision tree classifiers is that they can simultaneously employ several feature subsets and rules, as well as various levels of categorization. A significant decision tree consists of a root node, several inner and leaf nodes, and branches. A leaf node indicates the class given to the sample. Each internal node of the tree represents a feature, and the tree’s branches demonstrate the connections between features and classifications [21,22]. A decision tree classifier’s efficacy is influenced by how well the tree is constructed from the training data. A decision tree typically begins at the root node and divides the source phrase into subsets based on feature values to form subtrees. This procedure is repeated for each generated subset until a leaf node is produced. By examining data linked to fetal heart rate, fetal movement, and other physiological parameters, decision tree classifiers can be beneficial in diagnosing intrauterine fetal death. The model will be trained using a dataset of recorded cases of intrauterine fetal death and healthy pregnancies to find trends and generate predictions about new instances.

2. Random Forest Classifier

Bootstrapping and aggregation, or bagging, an ensemble technique, is utilized by the Random Forest Classifier to train multiple decision trees simultaneously. Bootstrapping uses various subsets of readily available characteristics to simultaneously train multiple decision trees on distinct subsets of the training sample. The Random Forest Classifier’s overall variance is decreased by bootstrapping to guarantee a unique decision tree in the random forest. Because it aggregates the evaluations of multiple trees for the final decision, the Random Forest Classifier has excellent generalization [21]. Without the risk of overfitting, the Random Forest Classifier outperforms most other classification algorithms in accuracy. Random Forest Classifiers can deal with complicated, non-linear correlations between input data and target variables. This is significant because numerous factors can lead to intrauterine fetal death, and these factors may interact in complicated ways. The Random Forest Classifier can detect these interactions and generate accurate predictions. Moreover, the Random Forest Classifier’s usage of several decision trees helps to limit the danger of overfitting the model to the training data, which can increase its generalization performance on new, unknown data.

3. KNN Classifier

KNN classifier KNN is a nonparametric classification approach. It is also a well-known classification algorithm. The basic idea is that known facts are placed in a space determined by selected features. When applied to an unknown set, the K training sets closest to the unknown set are examined via k-analysis NN’s of their pattern space. These k training sets accurately represent the unknown set’s KNN. Euclidean distance is used to define the closeness of sets. The most prevalent class among k-NNs in the unknown set is applied to the KNN classification. A smaller k value selects a training point closest to the test point and is more suitable for predicting the correct classification [22,23]. However, this estimate may be subject to high statistical variability due to the limited sample size. The KNN classifier can adapt to new data without needing to be retrained when dealing with intricate or changing datasets. In intrauterine fetal mortality, a KNN classifier can help discover patterns and anomalies in fetal heart rate data that human observers may miss. This can aid in the early detection and prevention of fetal distress, ultimately improving outcomes for both mother and child.

4. Gaussian Naïve Bayes

The Bayes theorem is used in the Naïve Bayes Classifier, a machine learning model. The Naïve Bayes Classifier [24] may determine the likelihood of the data input belonging to a specific class, represented as *A*, by monitoring the values of a specified set of attributes or parameters, denoted as *B*, in the equation.

(1) $$P\left(A|B\right)=\frac{P\left(B|A\right)P\left(A\right)}{P\left(B\right)},$$

where *P*(*B*/*A*) is the posterior probability and *P*(*A*) is the prior. Because it assumes that each variable is completely independent, the Naïve Bayes Classifier needs more training data to estimate the classification parameters. The continuous values of each class are typically assumed to be distributed normally (or Gaussian) when working with continuous data. The Gaussian Naïve Bayes model accepts continuous numerical features because they fit a Gaussian distribution. This classifier is especially useful when working with huge datasets, since it can swiftly assess and forecast based on the existing data. In the case of IUFD, the Naïve Bayes Classifier can assist in identifying patterns and risk factors that may not be immediately obvious to human observers, allowing for early intervention and improved results.

5. Adaboost Classifier

The Ensemble Approach AdaBoost trains and grows trees one at a time. AdaBoost makes use of boosting. The process of linking a sequence of weak classifiers so that each weak classifier seeks to improve the categorization of data misclassified by the weak classifier before it. The serial combination of weak classifiers accomplishes this to produce a strong classifier. Because decision trees employed in boosting techniques are biased rather than overfitted, they are referred to as “stumps.” One tree is trained to focus solely on the preceding tree’s flaws. Previous misclassified samples’ weights are raised such that subsequent trees focus on accurately categorizing previously misclassified data. Classification accuracy rises as the number of weak classifiers in the model grows. However, this may lead to severe overfitting and a reduction in generalization ability. AdaBoost works poorly with noisy data sets but well with skewed data sets. AdaBoost training requires time [22]. This is crucial when working with massive datasets since it may help reveal trends and anomalies that human observers may overlook. Adaboost can help detect risk factors and predictors of fetal distress in the context of intrauterine fetal mortality, allowing for earlier intervention and improved outcomes. Adaboost can assist in increasing prediction accuracy and reliability by merging numerous weak classifiers.

6. Gradient Boosting

Gradient Tree Boosting is a prediction technique that sequentially solves an infinite-dimensional optimization problem and generates a model in the form of a linear combination of decision trees. Gradient Boosting is a learning process that combines the results of a large number of elementary predictors to create a powerful committee that outperforms its individual members. Gradient Tree Boosting is a technique commonly used with fixed-size decision trees as base learners [25]. In the setting of intrauterine fetal death, allowing for early intervention and improved results. Gradient Boosting can enhance prediction accuracy and efficiency by integrating many decision trees into a single model.

7. Support Vector Classifier

The supervised machine learning method known as the Support Vector Classifier may tackle problems with both regression and classification. Using a training set of objects divided into classes, a hyperplane is located in the data space that offers the least minimum distance (referred to as margin) between items from various classes. As a result, the hyperplane is often referred to as the hyperplane with the greatest margin. Instead of using disparities in class means, Support Vector Classifier uses objects on the margin’s edges (support vectors) to divide objects. This is because the vectors closest to the margin support (define) the separating hyperplane [21]. In the case of intrauterine fetal death, early observation of these tendencies enables healthcare practitioners to intervene and take steps to mitigate detrimental consequences. Moreover, SVC can handle non-linear data, making it valuable in circumstances where other machine learning models could fail.

8. Voting Classifier

An output (class) that has the highest probability of becoming the result is predicted by a Voting Classifier, which trains on a large ensemble of models. The output class with the biggest majority of votes is estimated by compiling the results of each classifier that was supplied to the Voting Classifier. Instead of developing separate specialized models and assessing their accuracy, we propose a single model that takes into account two techniques, namely, KNN and Gradient Boosting, and estimates output based on their combined majority of votes for each output class [21]. Regarding intrauterine fetal death, the Voting Classifier can assist in identifying trends and risk factors, enabling early intervention and improved results. Further, merging multiple independent classifiers can increase prediction reliability and precision.

9. Feed Forward Network

This is a type of artificial neural network in which information travels in just one way, from the input layer to the output layer, via one or more hidden layers [26]. This network type is frequently used in machine learning applications such as classification tasks, where the aim is to assign a collection of input data to a given category or class. A Feed Forward Network may be trained on a dataset of multiple fetal health metrics, such as fetal heart rate variability, to predict the likelihood of fetal distress or other health concerns in the context of fetal health categorization. The use of a Feed Forward Network has a substantial influence on fetal health categorization. The application of machine learning techniques can aid in the reduction of diagnostic mistakes, the provision of more accurate evaluations of fetal health, and the ability of healthcare practitioners to make educated decisions regarding interventions and treatments. Finally, using a Feed Forward Network in fetal health categorization has the potential to enhance outcomes for both mother and baby by detecting potential health concerns early and allowing for prompt treatments.

#### 3.3.3. Cross-Validation Techniques

The number of samples that may be used to train a model is greatly decreased by splitting the critical data into three distinct sets, and the results can occasionally be influenced by randomization of the train, test, and validation sets.

1. K-fold Cross-Validation

For model selection and classifier error estimates, researchers frequently employ the K-fold Cross-Validation approach. A dataset is divided into k subsets using the K-fold Cross-Validation technique, and the learned model is then tested on the remaining subsets. In K-fold Cross-Validation, the initial sample is randomly split into k equal-sized subsamples. The remaining k subsamples are utilized as training data, while one of them is kept as validation data for evaluating the model. Each of the “k” subsamples is utilized precisely once as validation data after the cross-validation technique has been applied k times. The k estimates may then be averaged. The utilization of all samples for both training and validation distinguishes this strategy from repeated random subsampling, as well as the single validation of each observation. We have parametrized the k value as 10 [4].

2. Hold-Out Cross-Validation

In the Hold-Out method, data points are randomly assigned to two sets, generally referred to as the training examples and the test set. Each set’s size is arbitrary, but often, the test set is smaller than the training set. After that, the set is tested on test data and then trained using test data. In Cross Validation, the results of several model-testing runs are often averaged together; however, when applied alone, the Hold-Out strategy only comprises one run, which can occasionally provide erratic results compared to multiple runs.

3. Stratified K-fold Cross-Validation

Stratified folds are produced by the cross-validation class, a K-Fold variation. The folds are produced by maintaining a consistent proportion of observations for each class. This ensures that each dataset fold has the same percentage of instances with each label. It is, although, an improved version of the K-Fold method. When seeking to make inferences from multiple sub-groups or strata, stratified sampling is a typical sampling strategy. The data must not overlap, and the strata or sub-groups must be separate. As a basis, the Stratified K-Fold approach is favored over the K-Fold technique, which is used to solve classification problems with imbalanced class distributions. This Cross-Validation class produces stratified folds and is a K-Fold variation. The folds are generated by recording the proportion of observations in each class. We assigned the k value as 5 [27].

4. Leave-P-Out Cross-Validation

Cross-validation using the Leave-P-Out method uses P samples from the sample set as the test set and the remainder of the samples as the training set. The Leave-P-Out method requires n area samples, and takes $C_{n}^{p}$ times to train and test the model. The sample set is denoted by S. This is carried out again until the original sample is clipped on the training dataset and the validation data of p observations. We gathered 1000 random samples for testing and the rest for the training phase [28].

5. Leave-One-Out Cross-Validation

A subset of K-fold Cross Validation called Cross Validation with Leave-One-Out ensures that the number of folds matches the number of instances. Leave-One-Out Cross Validation should be employed when there are few instances of a class value in a data set to obtain a realistic accuracy estimate for a classification system. Because each fold has only one occurrence in Leave-One-Out Cross Validation, random partitioning is not required [29].

6. Monte Carlo

The dataset is randomly split into training and validation data using Monte Carlo Cross Validation. The model is fitted to the training instances for each such split, and the anticipated accuracy is determined using the validation data. The outcomes of the splits are then averaged. The advantage of this strategy is that the ratio of training to validation is unchanged by the number of repetitions. The drawback of this approach is that while certain observations might be selected more than once for the validation subsample, others might never be. In other words, subsets of validation would overlap. This strategy also displays Monte Carlo variation, which shows that the outcomes will be altered if the study is conducted with various random divisions. The model splits the data into 5 folds [30].

7. Repeated K-folds

A technique for raising a machine learning model’s expected performance is Repeated K-fold Cross-Validation. All that is needed is to repeatedly execute the Cross-Validation approach and provide the mean outcome across all folds from all runs. A high number of estimations is always desired to provide trustworthy performance estimation or comparison. Only k estimations are obtained in K-fold Cross-Validation. Running K-fold Cross-Validation many times is a standard way to increase the number of estimates. Before each round, the data is reshuffled and re-stratified. This mean result, as computed using the standard error, should be a more accurate representation of the model’s actual, underlying mean performance on the dataset. The model splits the data into 2 folds while iterating 2 times.

#### 3.3.4. Black-Box Evaluation

With the development of different machine learning algorithms, there is no proper interpretability from the models on how they achieve the prediction. Explainable AI was created to produce more explainable models while maintaining a higher level of learning performance. To understand each model’s performance, LIME and SHAP are applied to evaluate each model. LIME is a technique in which, by successfully approaching it with an interpretable model, it may effectively explain the outputs of any classifier or regressor. It is capable of selecting one of two classifiers. In practice, it generalizes well. An unreliable classifier trained on LIME that was used to undertake feature engineering on the given dataset can also be significantly enhanced [31]. SHAP provides a priority rating for each characteristic for each prediction. Its innovative components, the discovery of a new category of cumulative feature significance indicators, and simulated results demonstrating the existence of a different solution in this category with a range of desirable characteristics are all highlighted [32]. In Table 1, each model applies LIME to obtain the important features that determine the prediction for that model.

**Figure 8:**
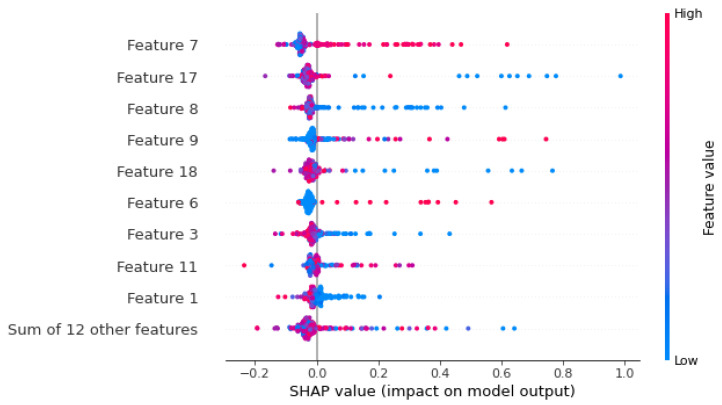
SHAP value analysis of features.

Prolonged Deceleration, Histogram Mode, Histogram Mean, Abnormal short-term variability, Baseline Value, Fetal Movement, Mean value of short-term variability, Mean value of short-term variability, and Severe Decelerations mainly determined the model output in Figure 8, while the SHAP value of each feature provided the impact on model output. Prolonged Deceleration played a huge impact on every model outcome for providing better prediction accuracy.

## 4. Results

This paper evaluated various machine learning algorithms before and after applying Cross-Validation techniques. The fetal cardiotocography dataset was randomly divided into 80% training and 20% test sets. Exploratory Data Analysis was conducted to derive the importance of the features. The working mechanism of each model with LIME and SHAP was explored, and the optimum model was achieved, with an accuracy of 0.99. The records taken for each class label are provided in Table 2. The impact of Cross-Fold Validation techniques can be seen from the performance results tabulated in Table 3 and Table 4. Table 3 holds the classifier performance before Cross-Fold Validation. Table 4 holds the classifier performance after Cross-Fold Validation measured using the performance metrics of accuracy, precision, recall, F1 score, Kappa, and MCC. Algorithm 1 shows the proposed work implementation steps.
**Algorithm 1** Proposed Methodology1:**START**2:Input: Input Fetal data record X{x1,x2,x3…}3:Output: Return the best-optimized model among other models4:Process
Standard Scaler: Standardize all input features for better evaluation using the formula$z=\frac{x-\mu }{\sigma }\;\;\;\;\;\forall \;x\;in\;X.$where *μ*: Mean, *σ*: Standard Deviation; *z*: standardized inputRandom Over Sampling is performed on minority classes—Suspect and PathologicalTrain–Test Split: with random state parameter = 10 and test size = 0.2Train every base classification model after data pre-processingThe output labels are defined as 1, 2, 31—Normal2—Suspect3—PathologicalTrain each model after applying different Cross-Validation techniques and evaluate it with performance metrics.FOR every base modelFOR every cross-validationTrain the model on training dataTest: Evaluating using Performance Metrics, i.e., Precision, Recall, F-1 Score, Kappa, and MCCChoose the optimized model after applying a particular cross-validation
5:**END**

The binary classification of fetal demise produces four outcomes—True positive, True negative, False positive, and False negative.

- True positive (*TP*)—Correct positive prediction
- False positive (*FP*)—Incorrect positive prediction
- True negative (*TN*)—Correct negative prediction
- False negative (*FN*)—Incorrect negative prediction

1. Accuracy

The proportion of samples properly identified by the model to the total number of samples is known as model prediction accuracy.

(2) $$Accuracy=\frac{TP+TN}{TP+TN+FP+FN}.$$

2. Precision

The model’s accuracy is measured by the ratio of successfully categorized positive values to all anticipated positive samples.

(3) $$Precision=\frac{TP}{TP+FP}.$$

3. Recall

The recall of a model is defined as the proportion of correctly predicted positive samples to all positive samples.

(4) $$Recall=\frac{TP}{TP+FN}.$$

4. F1-Score

The F1 score of the model determines the harmonic mean of Precision and Recall.

(5) $$F1-Score=\frac{Precision\cdot Recall}{Precision+Recall}.$$

5. Kappa

The kappa score, known as inter-rater reliability, evaluates the degree of agreement between real and estimated values.

(6) $$\kappa =\frac{2\left(TP\cdot TN-FN\cdot FP\right)}{\left(TP+FP\right)\left(FP+TN\right)+\left(TP+FN\right)\left(FN+TN\right)}.$$

6. Matthews Correlation Coefficient (*MCC*)

The Matthews Correlation Coefficient (*MCC*) is a model-evaluation tool. It calculates the difference between actual and projected values. It is used to assess the accuracy of binary classifications. True negatives, true positives, false negatives, and false positives are all considered by the coefficient. Only if the prediction delivers good values in all four of these areas does this trustworthy metric offer high scores.

(7) $$MCC=\frac{TN\cdot TP-FN\cdot FP}{\sqrt{\left(TP+FP\right)\left(TP+FN\right)\left(TN+FP\right)\left(TN+FN\right)}}.$$

## 5. Discussion

After the model underwent different Cross-Validation techniques, all models increased their accuracy by ~2% (Table 5). The Decision Tree Classifier increased from 0.92 to 0.96, the Random Forest classifier increased from 0.95 to 0.96, the KNN classifier increased from 0.91 to 0.92, Gaussian Naïve Bayes increased from 0.71 to 0.86, the Adaboost classifier increased from 0.92 to 0.93, Gradient Boosting increased from 0.96 to 0.99, SVC increased from 0.89 to 0.94, and Voting Classifier, which is an ensemble of KNN and Gradient Boosting, increased from 0.93 to 0.99. From the overall models, Gradient Boosting and Voting Classifier perform better in accuracy, precision, recall, F1 score, Kappa, and MCC. The classifiers excel in performance in terms of accuracy, with K-fold Cross Fold, Repeated K-fold, Monte Carlo, and Stratified Cross-Fold-Validation are Voting Classifier, and Gradient Boosting, Hold-Out, Leave-P-Out and Leave-One-Out work well with Gradient Boosting. The most compatible classifier for Stratified Cross Fold is Gaussian Naïve Bayes, for Repeated K-fold Voting Classifier and Gradient Boosting. Leave-One-Out Cross Fold works best with Decision Tree, Random Forest and KNN Classifier. Monte Carlo performs well with SVC. Table 6 provides the comparison of performance with other state-of-the-art models. Only the XGBoost accuracy matches our proposed model.

## 6. Conclusions and Future Work

For this study, we trained nine machine-learning algorithms on the Cardiotocography dataset. The top two models were then combined using the Voting Classifier approach to create the Ensemble Model. To improve model performance, we applied several cross-validation techniques [33,34,35]. Throughout the use of these techniques, we evaluated and analyzed all machine learning models using classification assessment metrics. Our experiments demonstrate that the Gradient Boosting and Voting Classifier outperformed other machine learning models in several Classification Model tests, with an accuracy rate of 0.99, a recall rate of 0.98, a precision rate of 0.96–0.98, and an F1 of ~0.97. Despite the small number of examples in the data, all eight machine learning models performed well in this study. However, we acknowledge that analysis and model performance could be improved with larger datasets containing more CTG reports. Moving forward, we plan to gather additional CTG data with more in-depth characteristics and develop a new model that can accurately predict the fetus’ status.

## Figures and Tables

**Table 1:** Correlation matrix on important fetal attributes.

	Accelerations	Prolonged Decelerations	Abnormal Short-Term Variability	Percentage of Time with Abnormal Long-Term Variability	Mean Value of Long-Term Variability	Fetal Health
Accelerations	1	−0.127748624	−0.27957	−0.37394	−0.14236	−0.36407
Prolonged Decelerations	−0.12775	1	0.04622	−0.13733	−0.22651	0.484859
Abnormal Short-term Variability	−0.27958	0.04622	1	0.459413	−0.31510	0.471191
Percentage of Time with Abnormal Long-term Variability	−0.37394	−0.13733	0.45941	1	−0.17111	0.426146
Mean Value of long-term Variability	−0.14236	−0.22651	−0.31510	−0.17111	1	−0.22679
Fetal Health	−0.36407	0.48485	0.47119	0.42614	−0.2268	1

**Table 2:** Determine the importance of features using LIME.

Model	Feature	Percentage of Importance
Decision Tree Classifier	Prolonged Deceleration	0.25
	Histogram Mean	0.21
	Histogram Minimum	0.18
	Acceleration	0.14
Random Forest Classifier	Prolonged Deceleration	0.13
	Histogram Mean	0.10
	Acceleration	0.05
	Histogram Median	0.05
KNN Classifier	Histogram Minimum	0.05
	Abnormal Short-term Variability	0.05
	Histogram Mode	0.03
	Histogram Median	0.02
Gaussian Naïve Bayes	Histogram Variance	0.27
	Acceleration	0.24
	Histogram Mean	0.12
	Histogram Mode	0.10
Adaboost Classifier	Prolonged Deceleration	0.41
	Acceleration	0.14
	Histogram Mode	0.08
Gradient Boosting	Histogram Mean	0.37
	Prolonged Deceleration	0.19
	Acceleration	0.15
	Histogram Mode	0.07
Support Vector Classifier	Histogram Variance	0.24
	Histogram Median	0.11
	Histogram Mode	0.09
	Histogram Mean	0.07
Feed Forward Network	Abnormal Short-term Variability	0.34
	Acceleration	0.22
	Histogram Minimum	0.12
	Histogram Mean	0.02
Voting Classifier	Abnormal Short-term Variability	0.04
	Acceleration	0.02
	Histogram Minimum	0.02
	Histogram Mean	0.02

**Table 3:** Number of cases in each class label.

Class Label	Count
Normal Condition	1655
Suspect Condition	295
Pathological Condition	176

**Table 4:** Evaluation result before Cross-Validation.

Model	Accuracy	Precision	Recall	F1 Score	Kappa	MCC
Decision Tree Classifier	0.92	0.896	0.85	0.8733	0.79	0.79
Random Forest Classifier	0.95	0.886	0.9233	0.9	0.84	0.84
KNN classifier	0.91	0.78	0.88	0.8233	0.72	0.73
Gaussian Naïve Bayes	0.71	0.72	0.62	0.6166	0.43	0.50
Adaboost Classifier	0.92	0.8933	0.8566	0.8733	0.77	0.77
Gradient Boosting	0.96	0.9266	0.9366	0.93	0.89	0.89
SVC	0.89	0.7533	0.83	0.78	0.65	0.66
Voting Classifier	0.93	0.81	0.91	0.85	0.78	0.78
Feed Forward Network	0.79	0.62	0.79	0.70	0.65	0.67

**Table 5:** Evaluation result after Cross-Validation.

Model	CV Technique	Accuracy	Precision	Recall	F1-Score	Kappa	MCC
Decision Tree Classifier	K-fold	0.96	0.90	0.90	0.90	0.86	0.86
	Stratified K-fold	0.95	0.91	0.92	0.92	0.86	0.86
	Hold-Out	0.93	0.92	0.87	0.90	0.82	0.82
	Leave-P-Out	0.72	0.47	0.73	0.50	0.30	0.33
	Leave-One-Out	0.99	0.98	0.98	0.98	0.99	0.99
	Repeated K-fold	0.93	0.89	0.88	0.88	0.80	0.80
	Monte Carlo	0.95	0.90	0.92	0.91	0.84	0.84
Random Forest Classifier	K-fold	0.96	0.90	0.95	0.92	0.88	0.89
	Stratified K-fold	0.95	0.88	0.94	0.91	0.85	0.86
	Hold-Out	0.94	0.89	0.91	0.90	0.82	0.83
	Leave-P-Out	0.75	0.48	0.77	0.49	0.30	0.38
	Leave-One-Out	0.99	0.99	0.99	0.99	0.99	0.99
	Repeated K-fold	0.94	0.86	0.91	0.88	0.82	0.82
	Monte Carlo	0.94	0.86	0.90	0.88	0.83	0.83
KNN Classifier	K-fold	0.92	0.81	0.87	0.83	0.76	0.77
	Stratified K-fold	0.92	0.81	0.90	0.85	0.75	0.76
	Hold-Out	0.91	0.78	0.88	0.83	0.71	0.73
	Leave-P-Out	0.71	0.43	0.73	0.44	0.21	0.26
	Leave-One-Out	0.99	0.99	0.99	0.99	0.80	0.80
	Repeated K-fold	0.90	0.78	0.85	0.81	0.71	0.71
	Monte Carlo	0.91	0.78	0.88	0.82	0.73	0.74
Gaussian Naïve Bayes	K-fold	0.81	0.77	0.72	0.72	0.63	0.61
	Stratified K-fold	0.86	0.79	0.78	0.76	0.65	0.67
	Hold-Out	0.85	0.77	0.72	0.73	0.64	0.66
	Leave-P-Out	0.78	0.89	0.61	0.65	0.40	0.42
	Leave one out	0.73	0.76	0.66	0.65	0.47	0.53
	Repeated K-fold	0.84	0.75	0.75	0.72	0.62	0.64
	Monte Carlo	0.74	0.78	0.69	0.67	0.49	0.55
Adaboost Classifier	K-fold	0.93	0.88	0.90	0.89	0.81	0.81
	Stratified K-fold	0.91	0.85	0.88	0.86	0.75	0.75
	Hold-Out	0.91	0.78	0.89	0.82	0.61	0.74
	Leave-P-Out	0.86	0.79	0.87	0.82	0.72	0.72
	Leave-One-Out	0.90	0.83	0.85	0.84	0.66	0.66
	Repeated K-fold	0.91	0.83	0.85	0.84	0.68	0.69
	Monte Carlo	0.92	0.85	0.88	0.86	0.67	0.68
Gradient Boosting	K-fold	0.99	0.96	0.99	0.98	0.96	0.96
	Stratified K-fold	0.99	0.98	0.98	0.98	0.96	0.96
	Hold-Out	0.96	0.93	0.92	0.93	0.83	0.88
	Leave-P-Out	0.99	0.98	0.98	0.98	0.95	0.95
	Leave-One-Out	0.99	0.98	0.98	0.98	0.94	0.94
	Repeated K-fold	0.99	0.99	0.98	0.99	0.93	0.93
	Monte Carlo	0.99	0.98	0.98	0.98	0.96	0.96
SVC	K-fold	0.94	0.79	0.91	0.83	0.78	0.79
	Stratified K-fold	0.92	0.81	0.92	0.86	0.78	0.78
	Hold-Out	0.89	0.75	0.83	0.78	0.84	0.66
	Leave-P-Out	0.88	0.76	0.88	0.81	0.72	0.72
	Leave-One-Out	0.92	0.80	0.89	0.84	0.75	0.76
	Repeated K-fold	0.93	0.80	0.90	0.84	0.77	0.77
	Monte Carlo	0.94	0.83	0.94	0.87	0.81	0.82
Voting Classifier	K-fold	0.98	0.96	0.98	0.97	0.95	0.95
	Stratified K-fold	0.98	0.95	0.98	0.97	0.94	0.94
	Hold-Out	0.93	0.81	0.91	0.85	0.78	0.78
	Leave-P-Out	0.97	0.95	0.98	0.97	0.94	0.94
	Leave-One-Out	0.98	0.96	0.98	0.97	0.96	0.96
	Repeated K-fold	0.99	0.97	0.99	0.98	0.96	0.96
	Monte Carlo	0.99	0.96	0.99	0.97	0.96	0.96
Feed Forward Network	K-fold	0.81	0.90	0.81	0.89	0.87	0.85
	Stratified K-fold	0.78	0.90	0.78	0.88	0.87	0.86
	Hold-Out	0.79	0.89	0.79	0.88	0.85	0.86
	Leave-P-Out	0.70	0.90	0.67	0.80	0.82	0.80
	Leave-One-Out	0.90	0.85	0.89	0.87	0.86	0.87
	Repeated K-fold	0.79	0.88	0.79	0.88	0.86	0.87
	Monte Carlo	0.80	0.90	0.80	0.89	0.85	0.83

**Table 6:** Comparison with other State-of-art models.

Models	Description	Accuracy (%)
Random Forest Classifier [1]	The paper implemented Z-Normal to standardize the data and applied only 10-fold Cross-Validation.	94.5
XG Boost [2]	Parameter optimization is conducted using Grid search CV, studied the minimum child weight and subsample ratio.	98
Blender Model [3]	Combined Gradient Boosting Classifier, CatBoost Classifier, Extreme Gradient Boosting with soft voting parameter, and Light Gradient Boosting Machine.	95.9
Random Forest Classifier with Leave one out CV [4]	RF pipeline applied hyperparameter Elasticnet, which is used for penalising the weights of the model.	81.3
XG Boost Classifier [5]	The researchers applied Classification based on the association (CBA)-M1/M2 algorithm.	93
IVY [6]	IVY is a feed-forward model which inputs time-lapsed video and outputs a confidence score.	93
Random forest Classifier [7]	The model applies nested Cross-Validation.	93
Light GBM [8]	The researchers use the LightGBM with Bayesian Optimisation and Gaussian process regression and apply K-fold Cross-Validation ensembling.	95.82
XG Boost Classifier [9]	The model applied Multiclass log loss for the early stopping method.	96.75
XG Boost Classifier [10]	The model applied Stratified K-fold CV.	84.2
Sparse Support Vector Machine [13]	The model penalizes the weight by imposing l1 norm.	75
XG Boost Classifier [14]	For the feature selection process, the researchers applied MOGA-CD, which follows the Genetic algorithm.	94
CNN [16]	The proposed CNN model consists of three convolutional layers with ReLU as the activation function and Fully connected layer.	94.3
Proposed Model	Monte Carlo, one of the CV techniques which applies shuffle splitting of the data, enhances Gradient Boosting and Voting Classifier.	99

## Data Availability

The data supporting this study’s findings are openly available on UCI Machine Learning Repository at [https://archive.ics.uci.edu/ml/datasets/cardiotocography] (accessed on 10 February 2023).
